# Supplementary figures and images for: Development of an immuno-wall device for the rapid and sensitive detection of EGFR mutations in tumor tissues resected from lung cancer patients
Source: PLoS One. 2020 Nov 16;15(11):e0241422. doi: 10.1371/journal.pone.0241422 (PMC7668601; doi:10.1371/journal.pone.0241422)

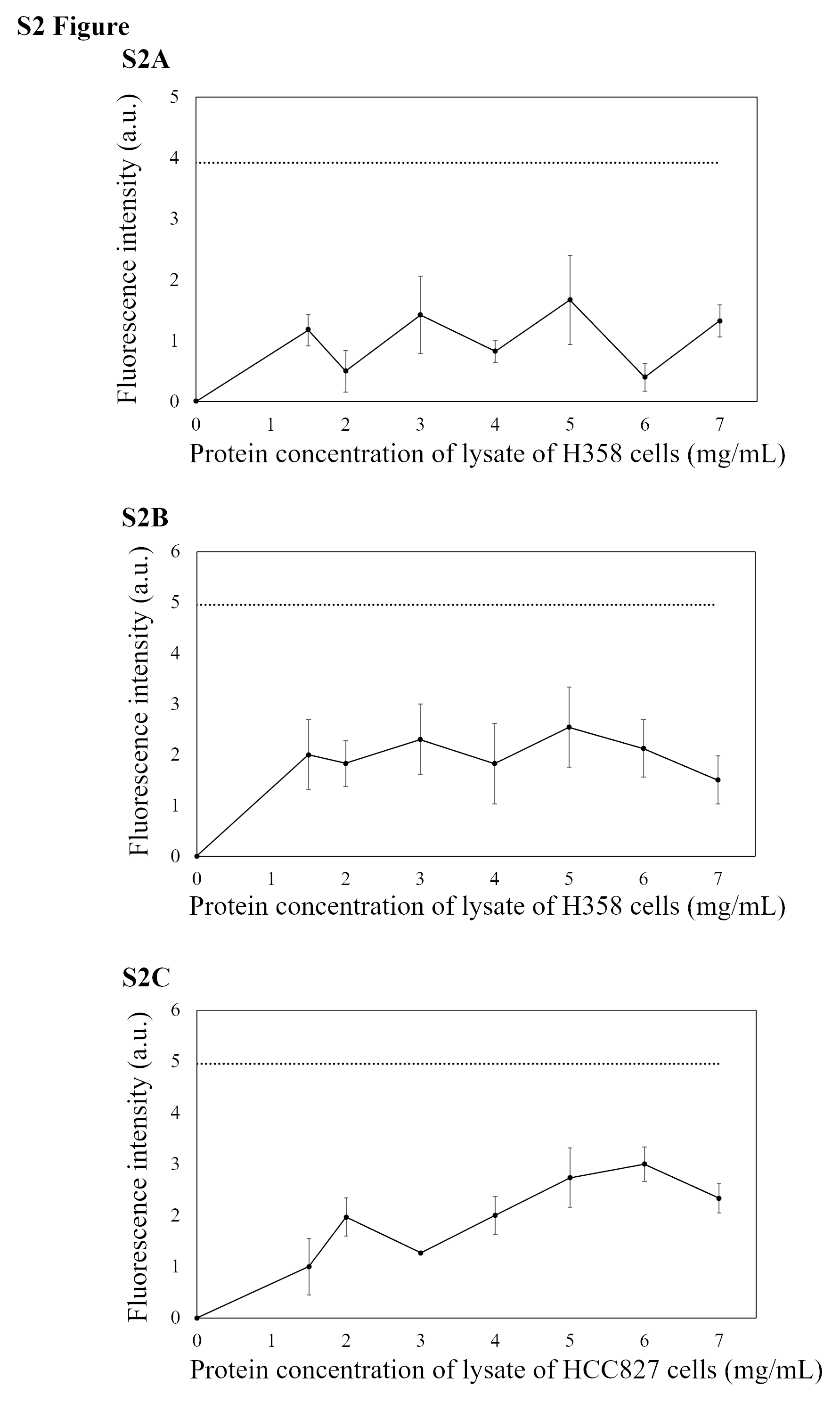

Supplement: S2 Fig — E746_A750 deletion for EGFR WT cell line (H358) (A), L858R substitution for EGFR WT cell line (H358) (B), and EGFR mutation cells (HCC827) (C). Dashed lines indicate three standard deviations above the average fluorescence determined in the EGFR WT cell line (H358) by each immuno-wall device. (TIF) [file pone.0241422.s002.tif]
