# Supplementary material for: Development of an immuno-wall device for the rapid and sensitive detection of EGFR mutations in tumor tissues resected from lung cancer patients
Source: PLoS One. 2020 Nov 16;15(11):e0241422. doi: 10.1371/journal.pone.0241422 (PMC7668601; doi:10.1371/journal.pone.0241422)
Supplement: S1 Table — (DOCX) [file pone.0241422.s005.docx]

S1 Table. Details of patients examined and immuno-wall analyses.

| No. | Age | Sex | Protein concentration of lysate (mg/mL) | DNA sequence for EGFR | Fluorescence intensity (a.u.) | | Immuno-wall analysis |
| --- | --- | --- | --- | --- | --- | --- | --- |
|  |  |  |  |  | E746_A750 del ^a^ | L858R ^b^ |  |
| 1 | 75 | M | 1.71 | wild | 1 | 3 | wild |
| 2 | 56 | M | 3.15 | E746_A750 del | 11 | 1.1 | E746_A750 del |
| 3 | 44 | M | 6.41 | E746_A750 del | 9 | 0.6 | E746_A750 del |
| 4 | 73 | F | 4.69 | L858R | 1 | 9 | L858R |
| 5 | 74 | M | 6.76 | wild | 1 | 2.7 | wild |
| 6 | 46 | F | 4.09 | wild | 1 | 1.4 | wild |
| 7 | 65 | M | 2.35 | wild | 1.2 | 1 | wild |
| 8 | 60 | M | 4.3 | wild | 2 | 1 | wild |
| 9 | 71 | M | 2.69 | wild | 1 | 3 | wild |
| 10 | 56 | F | 1.84 | wild | 2 | 1 | wild |
| 11 | 55 | F | 4.15 | E746_A750 del | 12.8 | 2 | E746_A750 del |
| 12 | 72 | M | 1.76 | E746_A750 del | 7.5 | 1 | E746_A750 del |
| 13 | 71 | M | 2.06 | E746_A750 del | 16 | 1 | E746_A750 del |
| 14 | 51 | F | 6.32 | E746_A750 del | 0 | 0 | wild |
| 15 | 75 | F | 2.3 | E746_A750 del | 6 | 0 | E746_A750 del |
| 16 | 70 | F | 2.42 | L858R | 0 | 2 | wild |
| 17 | 65 | M | 3.76 | L858R | 1 | 120 | L858R |
| 18 | 71 | F | 5.68 | L858R | 0 | 120 | L858R |
| 19 | 66 | F | 4 | L858R | 1 | 11.1 | L858R |
| 20 | 79 | F | 3.01 | L858R | 1 | 16.2 | L858R |
| 21 | 83 | F | 2.24 | L858R | 3.8 | 6 | L858R |
| 22 | 67 | F | 5.5 | L858R | 1 | 37.4 | L858R |
| 23 | 71 | F | 4.33 | E746_T751 del S752V P753S | 0 | 1 | wild |
| 24 | 72 | M | 2.69 | L747_E749 del A750P | 1 | 1.9 | wild |
| 25 | 65 | M | 4.34 | L747_E749 del A750P | 1 | 2 | wild |
| 26 | 68 | M | 3.48 | L747_A750 del T751P | 1 | 3.5 | wild |
| 27 | 79 | F | 4.33 | L747_T751 del | 1.7 | 1 | wild |
| 28 | 69 | F | 3.1 | L747_T751 del | 2 | 1.4 | wild |
| 29 | 73 | M | 2.54 | L747_T751 del | 1 | 1 | wild |
| 30 | 75 | F | 2.59 | L747_S752 del E746V | 1 | 1.4 | wild |
| 31 | 82 | F | 1.72 | L747_S752 del E746V | 0 | 1 | wild |
| 32 | 54 | F | 2.41 | L747_S752 del E746V | 2.5 | 1 | wild |
| 33 | 70 | F | 4.4 | L747_S752 delE746V | 0 | 1 | wild |
| 34 | 73 | F | 2.6 | L747_S752 del P753S | 1 | 2 | wild |
| 35 | 82 | M | 4.63 | L747_S752 del P753S | 1 | 1 | wild |
| 36 | 75 | F | 1.22 | L747_S752 del P753S | 1 | 2.4 | wild |
| 37 | 64 | F | 2.3 | L747_S752 del P753S | 1 | 1 | wild |

^a^ Fluorescence intensity higher than 3.92 a.u. was considered as positive

^b^ Fluorescence intensity higher than 4.95 a.u. was considered as positive
